# Knockdown-resistance (*kdr*) mutations in Indian *Aedes aegypti* populations: Lack of recombination among haplotypes bearing V1016G, F1534C, and F1534L *kdr* alleles

**DOI:** 10.1371/journal.pntd.0013126

**Published:** 2025-06-13

**Authors:** Taranjeet Kaur, Rajababu S. Kushwah, Sabyasachi Pradhan, Manoj K. Das, Madhavinadha P. Kona, Anushrita, Radhika Mittal, David Weetman, Rajnikant Dixit, Om P. Singh

**Affiliations:** 1 National Institute of Malaria Research, Dwarka, New Delhi, India; 2 National Institute of Malaria Research, Itki, Ranchi, India; 3 Liverpool School of Tropical Medicine & Hygiene, Liverpool, United Kingdom; CNRS: Centre National de la Recherche Scientifique, FRANCE

## Abstract

**Background:**

Knockdown resistance (*kdr*) mutations in the voltage-gated sodium channel (VGSC) gene are a key mechanism of insecticide resistance in mosquitoes. In Asian *Aedes aegypti* populations two main VGSC haplogroups with *kdr* mutations have been identified: one carrying the F1534C mutation and another with V1016G and/or S989P mutations. Previous functional studies have demonstrated that these three mutations on a single haplotype confer up to a 1100-fold increase in pyrethroid resistance, underscoring the importance of monitoring these triple mutations in distinct populations. This study investigates the prevalence of *kdr* mutations in Indian populations and explores the linkage association between these mutations and two distinct conserved types of introns located between exons 20 and 21.

**Methods:**

*Ae. aegypti* specimens collected from eight different locations were genotyped for *kdr* alleles and intron (between exons 20 and 21) haplotypes using PCR-based assays. Representative samples underwent DNA sequencing of VGSC regions.

**Results:**

Five *kdr* mutations namely S989P, V1016G, T1520I, F1534C, and F1534L were identified, each exhibiting varying distribution and frequencies across different geographical regions. Two distinct and stably-diverged intron haplotypes, designated as intron-A and intron-B, were identified between exons 20 and 21. Seven haplotypes, including two wild-type variants, were observed among Indian populations. The *kdr*-bearing haplotypes can be classified into three distinct haplogroups: haplogroup G (V1016G with/or without S989P and with intron-A), haplogroup L (F1534L and intron-A), and haplogroup C (F1534C with/or without T1520I and with intron-B). Importantly, no evidence of recombination within Indian populations was detected among these three haplogroups.

**Conclusions:**

Five *kdr* mutations were identified in the VGSC of Indian *Ae. aegypti* populations, each showing a definitive linkage with one of the two types of intron haplotypes. The lack of recombination among haplogroups bearing 1016G with 989P, 1534C and 1534L mutations suggests that the most potent insecticide resistance haplotype, bearing the triple *kdr* mutation, is currently absent. This finding has significant operational implications, as it may indicate that current vector control measures remain effective against these populations, potentially delaying the emergence of highly resistant phenotypes.

## Author summary

*Aedes aegypti* mosquitoes transmit dengue, chikungunya, and Zika, making their control crucial in India. Pyrethroid insecticides are widely used for mosquito control, but resistance is rising due to genetic mutations in the voltage-gated sodium channel (VGSC) gene. These mutations, known as knockdown resistance (*kdr*), reduce insecticide sensitivity and pose a growing challenge to vector control efforts. We analyzed *Ae. aegypti* from eight Indian regions to identify five key VGSC mutations—S989P, V1016G, T1520I, F1534C, and F1534L—and examined their association with two intron types (A and B) between exons 20 and 21. Our findings reveal distinct mutation patterns across India, forming three haplogroups with no evidence of recombination among them. Importantly, we did not detect the highly resistant triple mutation (989P + 1016G + 1534C) on a single haplotype, which has been associated with extreme pyrethroid resistance in other regions. These findings suggest that pyrethroids may still be effective in India for now, but the spread of resistant mutations highlights the need for continuous surveillance and alternative control strategies, such as insecticide rotation and integrated pest management, to sustain effective mosquito control and protect public health.

## Background

*Aedes aegypti* serves as a vector for several human arboviral infections including dengue virus (DENV), chikungunya virus (CHIKV), yellow fever virus (YFV), and Zika virus (ZIKV). Controlling these arboviral infections relies mainly on vector control measures. Pyrethroids, a commonly used group of insecticides for disease vector control, are favoured due to their low toxicity to mammals and rapid insect knockdown action. These insecticides act on insects by binding their open sodium channels and modifying the channel-gating kinetics by inhibiting the inactivation resulting in prolonged opening of the sodium channel and paralysis, and eventual insect death. Despite their efficacy, the effectiveness of pyrethroid-based control of *Ae. aegypti* is increasingly challenged by the escalating development of pyrethroid resistance worldwide [1], including in India [2, 3, 4]. Therefore, achieving effective management of resistance necessitates the ongoing monitoring of insecticide resistance patterns, a comprehensive understanding of the underlying mechanisms, and the identification of key markers for surveillance.

Knockdown resistance (*kdr*) is one of the mechanisms of insecticide resistance against DDT and pyrethroids which is due to the mutation/s in the voltage-gated sodium channel (VGSC) - the target site of action for DDT and pyrethroids. These mutations render the VGSC less susceptible to DDT and pyrethroids. The most commonly reported *kdr* mutation, L1014F/S, is not present in *Ae. aegypti* due to codon constraints [5], where a minimum of two SNPs are required in the codon to replace wild type Leucine with either resistant Phenylalanine or Serine amino acids. In *Ae. aegypti,* at least 23 mutations have been reported in the VGSC [6, 7, 8]. Among these, amino acid variants at the 1534 and 1016 VGSC residues are the most well-documented mutations, and their role in pyrethroid resistance has been functionally validated [9–10]. The distribution of *kdr* alleles show significant variation across different regions of the world. There are two alternative *kdr* mutations reported at residues V1016 (G/I) and F1534 (C/L), which exhibited distinct geographical distributions. Mutation V1016I is reported from the Americas, multiple African countries and Iran [11, 12, 13], while the alternative mutation V1016G (and linked mutation S989P) is reported from countries in Southeast Asia and Saudi Arabia [14] but has not been reported from countries in the Americas [11], except in Panama [15]. The T1520I is reported only from three Asian countries, India [7,16], Myanmar [17,18] and Laos [13].

Co-occurrence of multiple *kdr* mutations often with strong linkage associations have been reported, for example, S989P [3,13,14,19–21] and/or D1763Y [22,23] have been found in conjunction with V1016G while T1520I is associated with F1534C [3,18,24]. These linkages may have an additive or multiplicative effect on insecticide resistance levels [25,26] or may have a compensatory effect to overcome the deleterious effect of mutation on the fitness of the mosquito [3,27,28]. Linkage of *kdr* mutations has also been shown with two distinct intron-haplotypes linking exon 20 and 21 of the VGSC, named intron A and B [29, 30, 31]. Functional analysis of various sodium channel types by Hirata et al. [26], as expressed in *Xenopus* oocytes, revealed that 989P + 1016G and 1534C individually reduced their sensitivity to permethrin by 100- and 25-fold, respectively. However, a VGSC with the 989P + 1016G + 1534C triple mutation present on same haplotype, reduced sensitivity by 1100-fold against permethrin and by 90-fold against deltamethrin. Thus, a single recombination could lead to critical failure of a pyrethroid-based vector control programme; therefore monitoring the occurrence of the triple *kdr* mutation in populations is viewed as crucial [26]. The present study was undertaken to monitor *kdr* mutations in Indian populations and screen for the presence of triple mutant *kdr* mutations (989P + 1016G + 1534C), if any. In addition, the study also sought to establish linkage associations of different *kdr* mutations with the two intron types in Indian populations.

## Materials and methods

### Mosquito sampling

**Fig 1:**
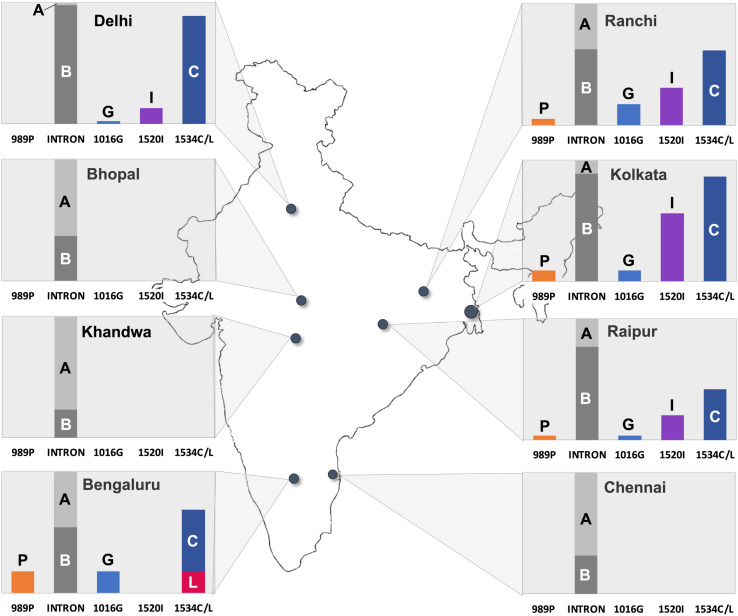
Frequencies of *kdr* mutant alleles (P = 989P, G = 1016G, I = 1520I, C = 1534C, L = 1534L) and intron types (A = intron type A, B = intron type B) in different populations of India. The base map of India was created by the corresponding author and was previously published in PLoS One (https://doi.org/10.1371/journal.pone.0253173). It is reused here with appropriate attribution.

*Aedes aegypti* immatures were collected from natural breeding habitats across various regions of India (Fig 1), namely New Delhi (28.58° N, 77.05° E), Bhopal, Madhya Pradesh (23.26° N, 77.41° E), Khandwa, Madhya Pradesh (21.83° N, 76.35° E), Ranchi, Jharkhand (23.35° N, 85.30° E), Raipur, Chhattisgarh (21.25° N, 81.63° E), Kolkata, West Bengal (22.57° N, 88.36° E), and Chennai, Tamil Nadu (13.08° N, 80.27° E). Late-stage larvae (III-IV instars) and pupae were sampled from multiple peri-domestic breeding sites, such as roadsides, parks, and other public spaces which are sparsely distributed. To minimize sampling bias and avoid over-representation of closely related individuals, no more than four immatures were collected per breeding site to enhance the likelihood of capturing a genetically diverse population and minimizing sampling bias. Collected immatures were transferred to plastic bowls containing water until pupation. Larvae were fed with a mixture of ground dog biscuit and yeast in a ratio of 3:1. Pupae were subsequently moved to bowls with water and placed in mosquito cages. Inside the mosquito cage, a cotton pad soaked in a 10% aqueous glucose solution served as a food source for the mosquitoes. Upon emergence, the mosquitoes were anesthetized using diethyl ether and identified morphologically under a stereo dissecting microscope. Morphologically confirmed *Ae. aegypti* specimens were stored individually in micro-centrifuge tubes, containing dehydrated silica gel wrapped in a small piece of paper. The collected mosquitoes were transported to the laboratory in Delhi and stored at -20 °C until DNA isolation.

### DNA isolation

Genomic DNA was isolated from individual mosquitoes using a salt-based precipitation method following the protocol of Black & Duteau [32]. Briefly, tissue was homogenized in a grinding buffer containing SDS, EDTA, Tris-HCl, NaCl, and sucrose, followed by mechanical disruption using a pestle. The homogenate was incubated at 65°C to enhance lysis and inactivate nucleases. 8M Potassium Acetate was added to precipitate SDS and contaminants, with subsequent centrifugation to remove debris. DNA was precipitated using cold ethanol, washed with 70% ethanol to remove residual salts, and resuspended in 200 μL of TE buffer. Prior to DNA isolation from female mosquitoes, the last three segments of the abdomen were excised to eliminate the spermatheca, which may contain sperms from male mating partners. The DNA was stored at 4 °C for immediate use or at -20 °C for prolonged storage. Additionally, DNA samples isolated from field-collected mosquitoes in Bengaluru in a previous study by Kushwah et al. [7] were also incorporated into this present study.

### Genotyping of *kdr* alleles

Genotyping of *kdr* alleles was conducted on DNA extracted from individual mosquitoes using PCR-based techniques developed by Kushwah et al. [3,7]. The list of primers is provided in Table 1. Briefly, for genotyping of domain III-*kdr* mutations, i.e., F1534C, F1534L and T1520I, a single PCR product was amplified using primers AekdrF and AekdrR followed by restriction digestion of the PCR product with three different restriction enzymes, i.e., *BsaB*I (for T1520I), *Ssi*I (for F1534C), and *Eco88*I (for F1534L)—each in separate 0.5 mL microcentrifuge tubes. For genotyping of S989P and V1016G *kdr* mutations, two independent allele-specific PCRs were performed using common flanking primers AedIIF and AedIIR along with two allele-specific primers, i.e., PPF and SSR for S989 alleles and VVF and GGR for V1016 alleles (Table 1) following the methodology described by Kushwah et al. [7]. All PCRs were carried out using Hot Start DNA polymerase (DreamTaq, ThermoFisher).

**Table 1:** Primers used for *kdr* genotyping, intron haplotype identification, and DNA sequencing.

Name of primers	Sequence (5´-3´)	References
**For *kdr* genotyping**		
AekdrF	TGGGAAAGCAGCCGATTC	Kushwah et al., [3]
AekdrR	CCTCCGTCATGAACATTTCC	Kushwah et al., [3]
AedIIF	AGACAATGTGGATCGCTTCC	Kushwah et al., [7]
AedIIR	GGACGCAATCTGGCTTGTTA	Kushwah et al., [7]
PPF	GGCGAGTGGATCGAAC	Kushwah et al., [7]
SSR	GCATACAATCCCACATGGA	Kushwah et al., [7]
VVF	TCCCACTCGCACAGGT	Kushwah et al., [7]
GGR	GGCTAAGAAAAGGTTAAGTC	Kushwah et al., [7]
**For intron haplotype identification**		
HapU_F	GTAAATTGGAGCGCACAACA	This study
HapA_R	ATGCGCGTCTAGTATTGCTG	This study
HapB_R	TGCTATCAAGATCAACGGTCTTT	This study
**For DNA sequencing**		
AedIIF	AGACAATGTGGATCGCTTCC	Kushwah et al., [7]
AedIIR	GGACGCAATCTGGCTTGTTA	Kushwah et al., [7]
AedIIIF	AACGTTCAAGGGCTGGATCC	Kushwah et al., [7]
AedIIIR	TTCGAGCCCATCTTTTTCAT	Kushwah et al., [7]

### Identification of intron-haplotype using PCR

**Fig 2:**
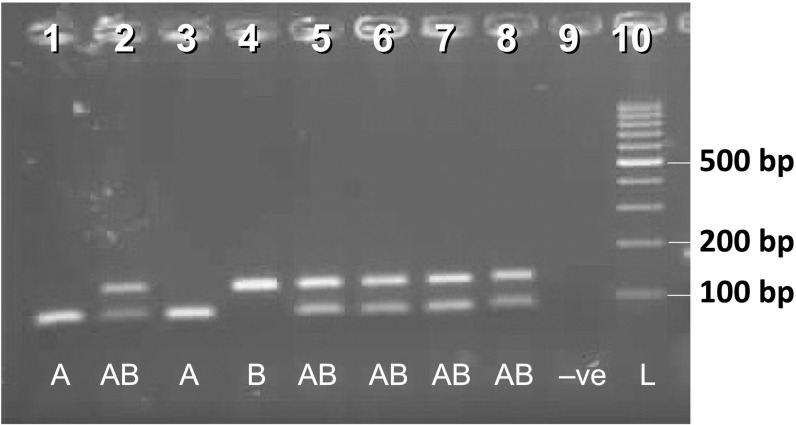
Gel photograph of PCR used for genotyping intron haplotypes. Samples in lanes 1, 4, 5 and 7 are molecularly confirmed through sanger sequencing for intron type. Abbreviations used: A = homozygous for intron A, B = homozygous for intron B, AB = heterozygous for introns A and B, -ve = negative control (no DNA), L = 100 bp DNA ladder.

As observed by earlier workers [23,30,33,34], two distinct intron haplotypes (connecting exons 20 and 21) were observed in domain II of the VGSC with a high degree of nucleotide and size polymorphism, which were named A and B following Martins et al. [30]. For the identification of intron types, a multiplex PCR strategy was developed. A universal forward primer HapU_F (Table 1) was designed from a conserved region of the intron. Two intron haplotype-specific reverse primers, HapA_R and HapB_R (Table 1), were designed which were specific for haplotypes A and B, respectively. The intron haplotype-specific primers were designed in such a way that at least five nucleotides at the 3’ end of each primer had mismatches with the non-target haplotype to ensure that there was no non-specific extension. Multiplex PCR amplification was performed using 2X DreamTaq Hot Start DNA polymerase (ThermoFisher) in a 15 µl reaction volume. Each reaction contained 0.2 µM of each primer, i.e., HapU_F, HapA_R and HapB_R. The thermal cycling program included an initial denaturation at 95 °C for 3 minutes, followed by 35 cycles consisting of denaturation at 95 °C for 15 seconds, annealing at 55 °C for 15 seconds, and extension at 72 °C for 30 seconds. A final extension was carried out at 72 °C for 7 minutes. PCR products were separated on a 3% agarose gel and visualized under UV light using a gel documentation system. The diagnostic amplicon sizes for haplotypes A and B were, 81 and 128 bp, respectively (Fig 2).

### Haplotype phasing

Estimation of haplotype definitions and frequencies based on genotyping results of loci S989, V1016, T1520, F1534 and intron types, was performed on pooled data using the Gibbs sampling strategy implemented in Arlequin ver 3.5 [35]. Since this method relies on homozygote data for reliable haplotype phasing, and some populations lacked homozygotes at certain loci, the data were pooled across populations for the analysis.

### Linkage disequilibrium analysis

Pairwise linkage disequilibrium (LD) analysis was performed between S989, intron, V1016, T1520, and F1534 loci using Fisher’s exact test to assess statistical significance and D’ values to measure the strength of LD. Genotypic data were pooled across populations and analyzed in R version 4.4.2 [36] using a contingency table-based approach. The dataset was expanded based on sample counts, ensuring accurate haplotype representation. Fisher’s Exact Test was performed using the fisher.test()function from the stats package [37], with Monte Carlo simulation (B = 100,000 permutations) to compute p-values for statistical significance. D’ values were calculated using haplotype frequency estimates derived from contingency tables, implemented in data.table for efficient data handling [38].

### DNA sequencing

Sequencing was performed on DNA samples isolated from individual mosquitoes for partial sections of domains II and III. The PCR amplifications for domain II were carried out using primers AedIIF and AedIIR, while for domain III, primers AedIIIF and AedIIIR from Table 1 were employed, following the method by Kushwah et al. [7]. PCR products underwent ExoSAP-IT (Thermo Fisher) treatment for the removal of primers and dNTPs and were sequenced using ABI BigDye Terminator v3.2 (ThermoFisher). The sequences were analyzed using Finch TV ver. 1.5.0. The number of samples sequenced for both domains II and III were 79 (majority homozygous for intron types) and 68 respectively.

## Results

Five *kdr* mutations were identified, S989P and V1016G in domain II, and T1520, F1534C, and F1534L in domain III of the VGSC, each exhibiting varying geographical distribution and frequencies (Fig 1 and Table 2). Mutations were recorded in a total of five populations, i.e., Delhi, Ranchi, Raipur, Kolkata and Bengaluru while no *kdr* mutations were found in three populations, i.e., Bhopal, Khandwa and Chennai. The most dominant mutation present in all five populations having *kdr* mutations was F1534C with allelic frequencies ranging from 42–89%. The second most common mutation was T1520I present in four populations, i.e., Delhi, Raipur, Ranchi, and Kolkata with allele frequencies ranging from 13–56%. The mutation V1016G was present in these five populations with low frequencies ranging from 2-18%. The mutation S989P was present in Ranchi, Raipur, Kolkata and Bengaluru with frequencies ranging from 2–18%. The mutation F1534L was only detected in Bengaluru, as reported previously [7].

**Table 2:** *kdr*-genotypes and intron types scored in different populations.

Locus	Attributes	Genotypes/ alleles	Localities							
**DEL**	**KOL**	**BPL**	**RNC**	**RAI**	**KND**	**BNG**	**CHE**
	n		156	240	162	57	54	204	561	182
S989	Genotypes	SS	156	203	162	52	52	204	388	182
		SP	0	31	0	4	2	0	148	0
		PP	0	6	0	1	0	0	25	0
	Allelic frequencies	S	1.00	0.91	1.00	0.95	0.98	1.00	0.82	1.00
		P	0.00	0.09	0.00	0.05	0.02	0.00	0.18	0.00
Intron	Intron type	AA	0	6	67	11	3	123	110	88
		AB	7	42	70	21	19	68	295	73
		BB	149	192	25	25	32	13	156	21
	Allelic frequencies	A	0.02	0.11	0.63	0.38	0.23	0.76	0.46	0.68
		B	0.98	0.89	0.37	0.62	0.77	0.24	0.54	0.32
V1016	Genotypes	VV	149	203	162	38	52	204	388	182
		VG	7	31	0	18	2	0	148	0
		GG	0	6	0	1	0	0	25	0
	Allelic frequencies	V	0.98	0.91	1.00	0.82	0.98	1.00	0.82	1.00
		G	0.02	0.09	0.00	0.18	0.02	0.00	0.18	0.00
T1520	Genotypes	TT	116	50	162	33	32	204	561	182
		TI	40	112	0	13	22	0	0	0
		II	0	78	0	11	0	0	0	0
	Allelic frequencies	T	0.87	0.44	1.00	0.69	0.80	1.00	1.00	1.00
		I	0.13	0.56	0.00	0.31	0.20	0.00	0.00	0.00
F1534	Genotypes	FF	0	7	162	12	20	204	72	182
		FC	35	51	0	20	23	0	169	0
		CC	121	182	0	25	11	0	134	0
		FL	0	0	0	0	0	0	39	0
		LC	0	0	0	0	0	0	139	0
		LL	0	0	0	0	0	0	8	0
	Allelic frequencies	F	0.11	0.14	1.00	0.39	0.58	1.00	0.31	1.00
		C	0.89	0.86	0.00	0.61	0.42	0.00	0.51	0.00
		L	0.00	0.00	0.00	0.00	0.00	0.00	0.17	0.00

Abbreviations used: n = number of samples tested, DEL = Delhi, KOL = Kolkata, BPL = Bhopal, RNC = Ranchi, RAI = Raipur, KND = Khandwa, BNG = Bengaluru, CHE = Chennai

DNA sequencing of domain IIS6 among the samples which were homozygous for specific intron haplotype alleles, revealed the presence of only two intron types, belonging to either intron-haplotype A and B. The two types of introns were highly conserved, as evidenced by comparison of the DNA sequences of 70 samples which were homozygotes for intron type (21 intron type A and 49 intron type B) from Ranchi, Kolkata, and Bengaluru with those from Fan et al. (2020) [39]. Intron A matched the DNA sequences of haplotypes V4 (S989/V1016), V8 (989P/1016G), and V9 (S989/1016I), while intron B was identical to haplotype V2 [39]. Genotyping of samples using intron-specific primers revealed that the proportion of these two intron haplotypes varied in different populations. The frequencies of intron-A ranged between 2 and 76% and of intron-B ranged between 24 and 98%. The proportion of intron B was highest in populations having *kdr* mutations (54–98%) while it was lowest in populations with no *kdr* (23–37%).

**Fig 3:**
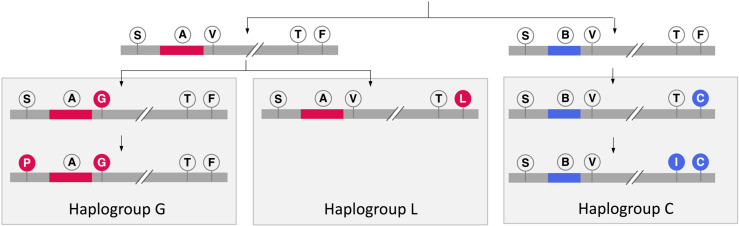
Hypothetical model illustrating the evolution of the three haplogroups (G, L, and C) based on *kdr* mutations and intron types. The 989P and 1520I mutations, occurring at lower frequencies than 1016G and 1534C, are assumed to have emerged later. (Abbreviation used: S = S989, P = 989P, A = intron type A, B = intron type B, V = V1016, G = 1016G, T = T1520, I = 1520I, F = F1534, C = 1534C, L = 1534L).

Phasing revealed the presence of a total of seven haplotypes including two wild haplotypes considering the polymorphisms at locus S989, intron type (connecting exon 20 and 21), V1016, T1520, and F1534. The number of samples with different genotype combinations including intron type in each population is shown in Table 3. The definition of haplotypes and their numbers is shown in Table 4. Of these, two wildtype haplotypes were SAVTF (n = 947) and SBVTF (n = 438), differing by their intron type (A vs. B). The five mutant haplotypes (i.e., those with at least one *kdr* mutation) included SBVT**C** (n = 1018), SBV**IC** (n = 365), **P**A**G**TF (n = 249), SAVT**L** (n = 194), and SA**G**TF (n = 21). The *kdr* alleles showed clear linkage association with other *kdr* alleles and intron types. The 1016G mutant was linked to intron-A and was present with or without 989P. The 1534C and 1534L mutants were linked to intron-B and former was present with or without 1520I. The five haplotypes with *kdr* allele(s) can be categorized into three haplogroups: (i) haplogroup G, with 1016G and/or 989P linked to intron type-A; (ii) haplogroup L, with 1534L linked to intron-A; and (iii) haplogroup C, with 1534C and/or 1520I linked to intron-B (Fig 3). The Fisher’s Exact Test revealed significant linkage disequilibrium (LD) across all loci pairs (p < 0.0001). The D’ values were consistently 1.0, indicating strong genetic linkage with minimal recombination between loci. This suggests that these loci are in complete LD, supporting their non-random association (S1 Table).

**Table 3:** Numbers of samples with different combinations of *kdr*-genotypes and intron types in different populations.

Genotype combinations^*^	Localities							
DEL	KOL	BPL	RNC	RAI	KND	BNG	CHE
PP AA GG TT FF	0	6	0	1	0	0	25	0
SP AA VG TT FF	0	0	0	0	1	0	31	0
SP AA VG TT FL	0	0	0	0	0	0	25	0
SP AB VG TI FC	0	19	0	2	0	0	0	0
SP AB VG TT FC	0	12	0	1	0	0	89	0
SP AB VG TT FF	0	0	0	1	1	0	3	0
SP AB VG TT FL	0	0	0	0	0	0	0	0
SP BB VG TT CC	0	0	0	0	0	0	0	0
SS AA VG TT FF	0	0	0	4	0	0	0	0
SS AA VV TT FF	0	0	67	6	2	123	12	88
SS AA VV TT FL	0	0	0	0	0	0	9	0
SS AA VV TT LL	0	0	0	0	0	0	8	0
SS AB VG TI FC	6	0	0	2	0	0	0	0
SS AB VG TT FC	1	0	0	8	0	0	0	0
SS AB VV TI FC	0	7	0	1	7	0	0	0
SS AB VV TT FC	0	4	0	6	2	0	58	0
SS AB VV TT FF	0	0	70	0	9	68	1	73
SS AB VV TT FL	0	0	0	0	0	0	5	0
SS AB VV TT LC	0	0	0	0	0	0	139	0
SS BB VV II CC	0	78	0	11	0	0	0	0
SS BB VV TI CC	22	83	0	8	7	0	0	0
SS BB VV TI FC	12	3	0	0	8	0	0	0
SS BB VV TT CC	99	21	0	6	4	0	134	0
SS BB VV TT FC	16	6	0	0	6	0	22	0
SS BB VV TT FF	0	1	25	0	7	13	0	21
Total	156	240	162	57	54	204	561	182

* order of loci: S989P; intron A/B; V1016G; T1520I; F1534C/L

Abbreviations used: DEL = Delhi; KOL = Kolkata; BPL = Bhopal; RNC = Ranchi; RAI = Raipur; KND = Khandwa; BNG = Bengaluru; CHE = Chennai.

**Table 4:** Haplotype definitions and their frequencies based on the Gibbs sampling strategy.

S. No.	Haplotypes	Total
1	SBVTC	1018
2	SAVTF	947
3	SBVTF	438
4	SBVIC	365
5	PAGTF	249
6	SAVTL	194
7	SAGTF	21
	Total	3232

Interestingly, the F1534C mutation, which is linked to intron-B, exhibits a markedly high frequency among intron-B haplotypes. In Kolkata, Ranchi, Delhi, and Bengaluru, the frequency of F1534C among these haplotypes is 97%, 99%, 91%, and 95%, respectively, suggesting that this mutation is nearing fixation in these populations. Notably, in Delhi, the V1016G mutation (without S989P) was recorded for the first time at a low frequency (2%), exclusively linked with the intron-A. Remarkably, all instances of intron-A carried the V1016G mutation, demonstrating a 100% allelic frequency within this haplotype group.

## Discussion

This study provides a comprehensive analysis of *kdr* mutations in *Ae. aegypti* populations across different regions of India, with an emphasis on understanding the prevalence and distribution of these mutations and their linkage association including intron haplotypes. The key findings indicate the presence of five *kdr* mutations (S989P, V1016G, T1520I, F1534C, and F1534L) with varying geographical distributions and frequencies. Furthermore, two distinct and conserved intron haplotypes (A and B) were identified between exons 20 and 21 of the voltage-gated sodium channel (VGSC) gene, which show strong linkage associations between specific *kdr* mutations and intron haplotypes in Indian *Ae. aegypti* populations.

Our findings highlight important geographical variations in the distribution of *kdr* mutations in Indian *Ae. aegypti* populations. The most prevalent mutation, F1534C, was detected across multiple locations, including Delhi, Ranchi, Raipur, Kolkata, and Bengaluru, with allelic frequencies ranging from 51% to 89%. The T1520I mutation, which is linked with F1534C, was present in all these localities where F1534C is prevalent, except Bengaluru, suggesting potential localized selection pressures or founder effects. The V1016G mutation, widely reported in Southeast Asian countries, was found at low frequencies in five Indian populations (Ranchi, Raipur, Kolkata, Bengaluru and Delhi) with the S989P mutation, typically associated with V1016G, except in Delhi.

The S989P and V1016G mutations were consistently linked with intron type A, while the F1534C mutation was linked with intron type B. This finding is consistent with earlier studies from other Asian countries, where similar associations have been documented [8,11,31,39]. We identified three major haplogroups based on *kdr* mutations and intron types, i.e., haplogroup-G (V1016G with/or without S989P and with intron-A), -L (F1534L and intron-A), and -C (F1534C with/or without T1520I and with intron-B). The absence of recombination among the three haplogroups in Indian populations suggests that these mutations are maintained within distinct genetic backgrounds, which could have important implications for the spread of high-level insecticide resistance.

The co-occurrence of 989P, 1016G and 1534C mutations is very common in Asian countries [6. 8, 40, 41] but 1016G (with or without 989P) and 1534C tend to be present on different haplotypes. Global genomic analyses [42] further support our findings that *kdr* mutations in *Ae. aegypti* exhibit strong linkage disequilibrium and independent selective sweeps, preventing the widespread co-inheritance of V1016G and F1534C on the same haplotype. This is likely due to localized selection pressures that shape resistance evolution in different regions. The presence of these *kdr* mutations, i.e., 989P + 1016G + 1534C on same haplotype has been shown to reduce sensitivity to permethrin by 1100-fold and to deltamethrin by 90-fold with resultant potential that a single recombination could result in extreme resistance [26]. This underscores the potential challenges in controlling *Aedes aegypti* populations in India using pyrethroid-based interventions. However, the absence of triple mutations in our study could suggest that while significant resistance is present, it may not yet be at the critical level that would compromise current vector control strategies. Several studies have reported co-occurrence of these triple mutations (989P + 1016G + 1534C) on the same chromosome in Myanmar [40], China-Indonesia border [43], Saudi Arabia [14,44–46], Malaysia [41] and Indonesia [47] in few individuals. In Beijing, China, however, all the three *Ae. aegypti* mosquitoes collected in a study, were homozygous for 989P and 1016G and heterozygous for 1534C [48]. Kasai et al. [8], also observed relatively high frequencies of V1016G + F1534C mutations (with/without S898P) on single haplotypes in various populations: 4.1% in Hanoi, 11.8-16.5% in Dak Lak, and 18.6% in Phnom Penh. In a study conducted in Myanmar [18], high frequencies of haplotypes carrying the 989P + 1016G + 1534C mutations (17–57%) were recorded across different localities, along with lower frequencies of haplotypes carrying 989P + 1016G + 1534L (2%) and 989P + 1016G + 1520I + 1534C (1%). Identifying intron types would be useful to determine whether the haplotype with the 1534C + 1016G mutations is due to the independent origin of either mutation or is a result of recombination. Overall, these findings indicate different evolutionary pressures or gene flow dynamics between these regions.

The asynchronous spread of *kdr* mutations across *Ae. aegypti* populations worldwide, as documented by Schmidt et al. (2024) [42], highlights the role of local selection pressures and gene flow in shaping resistance evolution. This may explain the newly emerging presence of V1016G in Delhi, despite its previous absence in earlier studies. The absence of recombination between the major haplogroups (G, L, and C) bearing *kdr* alleles in Indian populations further suggests that these mutations have been maintained separately, potentially due to strong linkage disequilibrium or limited genetic exchange. The absence of triple mutations (989P, 1016G and 1534C on a single haplotype) in Indian populations in this report aligns with a similar pattern observed in multiple regions such as Taiwan [23] India [7] Mauritania [49] China-Myanmar border [43] Malaysia [50,51] and Papua New Guinea [52]. These mutations commonly co-occur within populations but are not explicitly reported on a single haplotype and inference is based on the absence of homozygous triple mutants, suggesting 989P + 1016G and F1534C typically reside on separate haplotypes.

The geographic variation in these *kdr* mutations across India likely reflects a mix of local selection and gene flow. The high F1534C frequency (51–89%) and its near-fixation in intron-B haplotypes (91–99% in Delhi, Kolkata, Ranchi, Bengaluru) suggest local adaptation driven by pyrethroid use, consistent with global selection trends [11]. In contrast, V1016G’s low frequency (0–18%) in India, suggest a recent introduction, possibly via gene flow from neighbouring Myanmar, where it occurs at high frequencies (79–84%) [40]. F1534L’s restriction to Bengaluru (17%) and T1520I’s patchy distribution (13–56% in Delhi, Ranchi, Raipur, Kolkata); also Myanmar [17–18] and Laos [13] at low frequency, could stem from founder effects or localized selection pressures. These patterns highlight how India’s *kdr* diversity may arise from both resident adaptation and external introductions.

Earlier records shows the absence of V1016G *kdr*-mutation in Delhi [3], yet in this study, we detected it in seven heterozygous samples (six with 1534C and one with F1534) each without 989P from Delhi having this mutation in heterozygous condition (six with 1534C and one with F1534). This emerging presence, alongside intron-A in heterozygous condition (frequency 2%), suggests a recent introduction, potentially linked to the gene flow inferred above. All other Delhi samples exhibited intron-B (98% frequency), reinforcing F1534C’s dominance.

A review of the literature reveals consistent patterns in the co-occurrence of specific mutations within certain haplotypes. Notably, the T1520I mutation is invariably found alongside the F1534C mutation, indicating that they reside on the same haplotype. However, T1520I is not widespread; it was first reported in India [3] and has since been detected in Myanmar [17,18] and Laos [13]. In this study, we recorded this mutation in Delhi and Kolkata, always in conjunction with F1534C, but at low frequencies compared to F1534C. Similarly, the S989P mutation consistently co-occurs with the V1016G mutation across various studies whereas V1016G mutation occurred both independently and in combination with the S989P mutation. In several Asian countries, these mutations are typically linked, either occurring at similar frequencies [19,53,54] or with S989P present at a lower frequency relative to V1016G [40,43,50–52]. Rarely, V1016G has been reported to occur independently of S989P [8,55]. In our study, we identified V1016G without S989P exclusively in Delhi, where its frequency was extremely low (2%). Notably, a previous study [3] did not report the presence of V1016G in Delhi, suggesting a recent introduction of this mutation. In contrast, in Bengaluru, V1016G and S989P were found at equal frequencies, while in Ranchi, S989P occurred at a significantly lower frequency than V1016G. These findings highlight both regional consistency and variability in mutation linkage, suggesting that differences in evolutionary pressures or genetic backgrounds across populations may be influencing these patterns.

The mutations S989P, V1016G/I, and I1101M are typically linked with intron type A, while F1534C is generally associated with intron type B [23,29,44]. Our findings align with this pattern; however, in Ghana, the F1534C *kdr* mutation in *Ae. aegypti* has been found to be associated with intron type A [23]. In the study by Fan et al. [39] it was observed that the F1534C mutation appeared in at least two distinct haplotypes of the VGSC gene, corresponding to different clades (intron types A and B) and geographic locations. Cosme et al. [11] further confirmed that F1534C has arisen independently in multiple populations, with haplotype network analysis identifying at least two distinct evolutionary origins. This suggests that the F1534C mutation has two independent origins, likely due to separate selective pressures and subsequent spread in different populations. Recent genomic analysis [42] confirms two distinct evolutionary origins of the F1534C mutation in *Ae. aegypti*: one originating in North & South America, New Caledonia, Saudi Arabia, and Malaysia, and another found in Kenya, Thailand, Malaysia, Fiji, Tonga, and Kiribati. These findings suggest that the mutation arose independently in at least two genetic backgrounds and subsequently spread across geographically distant populations through selection and gene flow. Therefore, it is worth investigating whether the occurrence of the triple mutation (989P + 1016G + 1534C) on the same haplotype is due to recombination between haplotypes 989P + 1016G + intron-A + F1534 and S989 + V1016 + intron-B + 1534C, or if it is a result of an independent origin of F1534C. Genotyping intron types in conjunction with *kdr* genotypes could provide valuable insights into the evolutionary history of these mutations. Identifying intron types using DNA sequencing is limited by the inability to resolve heterozygous sequences due to the presence of indels, which causes sequence collapse. The PCR method developed in this study for genotyping intron haplotypes can help address this issue. Also, it is crucial to sequence the wider genomic region encompassing the loci S989P, V1016G, and F1534C/L, either by amplifying through long-read PCR [56] or using single molecule next-generation sequencing. Via this approach genetic signatures of recombination events, particularly within intronic regions where recombination hotspots are predominantly located [57,58] can be identified.

In this study, sequencing a limited number of samples identified two distinct intron types, A and B. Despite their divergence, each intron type remained highly conserved, with no observed variability, regardless of the presence or absence of *kdr* mutations. Intron-A matched the sequences found in haplotypes V4, V8, and V9 (have identical intron), while intron-B matched to V2 haplotype, as defined by Fan et al. [39]. Fan et al. [39] reported that the 989P and 1016G mutations are linked to intron-A, which is consistent with our findings. They also observed an association between the 1534C mutation and V2 (intron-B) in Asian populations, similar to our results, with the exception of one sample (out of 174 samples, excluding those from the Americas and Africa).

The F1534C mutation was found exclusively in locations where intron type B was the predominant haplotype. Furthermore, in several areas, including Kolkata, Ranchi, Delhi, and Bengaluru, the F1534C mutation is nearly fixed among intron-B haplotypes, with frequencies ranging from 91% to 99%. In contrast, a similarly high frequency of the 1016G mutation among intron-A haplotypes was observed only in Delhi, where all seven samples with intron-A (in heterozygous condition) carried this mutation.

This study has certain limitations. The geographic coverage was restricted to specific regions, and further research is needed to evaluate the prevalence of *kdr* mutations in other parts of India. Additionally, functional studies on mutations like S989P and T1520I that are linked to V1016G and F1534C, respectively, are needed to explore the potential compensatory effects of these mutations on mosquito fitness or their additive role in conferring resistance phenotype, which could provide further insights into the evolutionary dynamics of resistance. Finally, there is a need to investigate other resistance mechanisms. Furthermore, our study concentrated on characterizing the prevalence and linkage associations of five *kdr* mutations—S989P, V1016G, T1520I, F1534C, and F1534L—within domains II and III of the VGSC, which are well-documented in Indian *Ae. aegypti* populations and prevalent in other Asian countries. However, we did not explore mutations in other VGSC regions reported in various parts of the world. Future studies could broaden genotyping efforts to include all VGSC mutations documented globally, providing a more comprehensive understanding of resistance mechanisms and their evolutionary dynamics in Indian populations. The results presented here are based on immature collections. However, despite targeting sparsely distributed public sites, some individuals from the same container may have been siblings. This limitation could lead to a slight local alteration in mutation frequencies. Nevertheless, given the large sample sizes per population and the restricted number of larvae collected per container, the overall impact on our findings is expected to be minimal.

In conclusion, our study provides a detailed characterization of *kdr* mutations and their genetic associations in Indian *Ae. aegypti* populations. While integrated vector management approaches, combining chemical, biological, and environmental control methods, are necessary, an immediate priority should be to use of pyrethroids cautiously in populations where *kdr* mutations are prevalent. In areas without documented *kdr* mutations, resistance profiling should be conducted to assess whether pyrethroids might still be a viable option in the short term. Continued surveillance and targeted interventions will be essential for maintaining the effectiveness of vector control programs and reducing the burden of arboviral diseases in India.

## Supporting information

S1 Table Pairwise Linkage Disequilibrium (LD) analysis between S989, Intron, V1016, T1520, and F1534 loci. Fisher’s exact test p-values are shown in the upper diagonal, and D’ values are in the lower diagonal. (DOCX)
