# Supplementary material for: Knockdown-resistance (kdr) mutations in Indian Aedes aegypti populations: Lack of recombination among haplotypes bearing V1016G, F1534C, and F1534L kdr alleles
Source: PLoS Negl Trop Dis. 2025 Jun 13;19(6):e0013126. doi: 10.1371/journal.pntd.0013126 (PMC12165380; doi:10.1371/journal.pntd.0013126)
Supplement: S1 Table — (DOCX) [file pntd.0013126.s001.docx]

Table S1. Pairwise Linkage Disequilibrium (LD) analysis between S989, Intron, V1016, T1520, and F1534 loci. Fisher's exact test p-values are shown in the upper diagonal, and D' values are in the lower diagonal.

|  | S989 | Intron | V1016 | T1520 | F1534 |
| --- | --- | --- | --- | --- | --- |
| S989 | - | 1.00E-05 | 1.00E-05 | 3.00E-05 | 1.00E-05 |
| Intron | 1 | - | 1.00E-05 | 1.00E-05 | 1.00E-05 |
| V1016 | 1 | 1 | - | 3.00E-05 | 1.00E-05 |
| T1520 | 1 | 1 | 1 | - | 1.00E-05 |
| F1534 | 1 | 1 | 1 | 1 | - |
